# Patient and Public Involvement for Dementia Research in Low- and Middle-Income Countries: Developing Capacity and Capability in South Asia

**DOI:** 10.3389/fneur.2021.637000

**Published:** 2021-03-23

**Authors:** Jahanara Miah, Saima Sheikh, Rachel C. Francis, Gayathri Nagarajan, Sojan Antony, Maryam Tahir, Rabia Sattar, Anum Naz, Sehrish Tofique, Mostazir Billah, Sajib Saha, Iracema Leroi

**Affiliations:** ^1^Division of Neuroscience and Experimental Psychology, University of Manchester, Manchester, United Kingdom; ^2^Department of Speech Language Pathology, All India Institute of Speech & Hearing, Mysuru, India; ^3^Dementia Care in SCARF – DEMCARES, Chennai, India; ^4^Department of Psychiatric Social Work, National Institute of Mental Health and Neurosciences, Bengluru, India; ^5^Division for Neurocognitive Disorder, Pakistan Institute of Living & Learning, Karachi, Pakistan; ^6^Hearing Care Center Ltd., Renaissance Hospital & Research Institute, Dhaka, Bangladesh; ^7^School of Medicine, Global Brain Health Institute, Trinity College, Dublin, Ireland

**Keywords:** patient and public involvement, dementia research, co-production, co-creation, low- and middle-income countries, capacity and capability, public engagement onion model

## Abstract

**Background:** Patient and public involvement (PPI) is an active partnership between the public and researchers in the research process. In dementia research, PPI ensures that the perspectives of the person with “lived experience” of dementia are considered. To date, in many lower- and middle-income countries (LMIC), where dementia research is still developing, PPI is not well-known nor regularly undertaken. Thus, here, we describe PPI activities undertaken in seven research sites across South Asia as exemplars of introducing PPI into dementia research for the first time.

**Objective:** Through a range of PPI exemplar activities, our objectives were to: (1) inform the feasibility of a dementia-related study; and (2) develop capacity and capability for PPI for dementia research in South Asia.

**Methods:** Our approach had two parts. Part 1 involved co-developing new PPI groups at seven clinical research sites in India, Pakistan and Bangladesh to undertake different PPI activities. Mapping onto different “rings” of the Wellcome Trust's “Public Engagement Onion” model. The PPI activities included planning for public engagement events, consultation on the study protocol and conduct, the adaptation of a study screening checklist, development and delivery of dementia training for professionals, and a dementia training programme for public contributors. Part 2 involved an online survey with local researchers to gain insight on their experience of applying PPI in dementia research.

**Results:** Overall, capacity and capability to include PPI in dementia research was significantly enhanced across the sites. Researchers reported that engaging in PPI activities had enhanced their understanding of dementia research and increased the meaningfulness of the work. Moreover, each site reported their own PPI activity-related outcomes, including: (1) changes in attitudes and behavior to dementia and research involvement; (2) best methods to inform participants about the dementia study; (3) increased opportunities to share knowledge and study outcomes; and (4) adaptations to the study protocol through co-production.

**Conclusions:** Introducing PPI for dementia research in LMIC settings, using a range of activity types is important for meaningful and impactful dementia research. To our knowledge, this is the first example of PPI for dementia research in South Asia.

## Introduction

Associated with population aging, dementia is emerging as an increasingly prevalent condition, particularly in low- and middle-income countries (LMIC) where about two-thirds of the world's population with dementia reside (1). In South Asia alone, the proportion of people living with dementia is estimated as 5.1 million (2). Health and social care services for this population, or for older people in general, is limited (3) or, in many areas, non-existent. Thus, developing such services, guided by locally obtained evidence, is a priority; however, in many LMICs, research capability for non-communicable diseases (NCD) in general is still developing (4), and for dementia research, this situation is magnified. Thus, building capacity and capability to conduct dementia research is essential (5), and is aligned with the priorities outlined in the 2019 position statement, “*Roadmap for Dementia Research in Pakistan”* developed by an international group of expert stakeholders interested in dementia research in South Asia (6).

The involvement of people with the lived experience of a health condition, and their families, “Patient and Public Involvement” (PPI) is a cornerstone of any applied research, particularly involving international collaborations where cultural adaptation of interventions and methods to local contexts is required. PPI is well-established in several high-income countries, particularly the United Kingdom, Australia, and Canada (7–9) and is viewed as an active involvement characterized in the form of consultation, collaboration or user control (10, 11). However, in many LMICs, the concept and practice of PPI for both research and service development is not well-known (12, 13), and is potentially challenging due to the established patient-professional hierarchical structures prevalent in many LMIC health systems.

The common ethos of PPI is defined as “research being carried out ‘with’ or ‘by’ patients and public members rather than ‘to’, ‘about’ or ‘for’ them” (10, 14). It recognizes the centrality of the patient and public's viewpoints and concerns, and the acknowledgment that their perspective may differ from those of researchers (15–17). In under-resourced LMIC settings, the theoretical underpinnings of PPI (18–21) take on greater significance including, the following, as conceptualized by Greenhalgh et al. (22): (1) the “*emancipatory imperative*”, which suggests that involving people in research addresses power imbalances between participants, who may be vulnerable populations, and researchers, and encompasses the social justice principle of “inclusion”; (2) the “*efficiency imperative”*, which addresses the need to reduce “research waste” (23, 24) by addressing critical research questions pertinent to the population in question, and the need to accelerate the research trajectory from “proof-of-principle” to implementation (16, 17, 21, 25); and (3) the “*political imperative”* which holds that knowledge should be co-created by researchers and lay stakeholders (26–29). In addition, if the research involves international partners, particularly those from HIC, all three imperatives are important, particularly to safeguard against the risk of “research imperialism” (30).

An additional and important driver to undertake PPI is the need to provide a platform for people with dementia (PwD) and their care partners to communicate their experiences and to have an influence on research (8, 11, 23, 26, 28, 31–37). Recently, increased accessibility through technology is providing greater opportunities for people with dementia to be empowered and to have their voices “heard”; however, in many LMIC settings access to such technology may still be limited, particularly for older people such as those living with dementia and their care partners.

**Figure 1:**
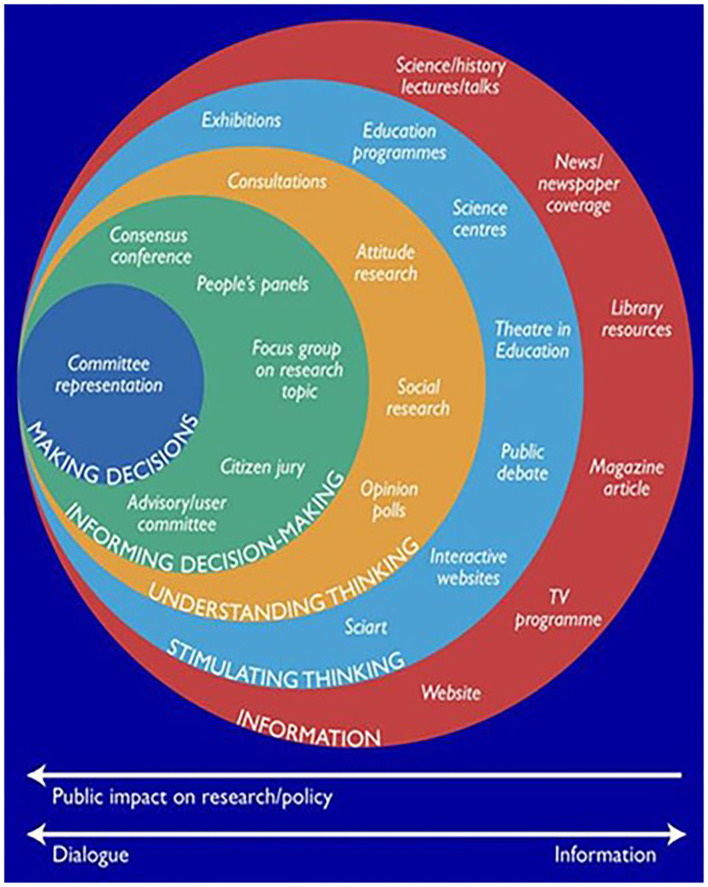
The “Public Engagement Onion” model (41) (Image courtesy of the Wellcome Trust).

There is, therefore, a need to strengthen the capacity of PPI in LMIC, particularly for newly emerging areas of research and practice, such as dementia care (6). Furthermore, PPI in LMIC settings plays a pivotal role in cultural adaptation of interventions where the language, cultural practices, context and health literacy may be limited (38). Here, we have synthesized our learning and reflection on PPI capacity building in LMIC (22), developed in the context of a dementia-related feasibility study in seven sites across three South Asian countries: Bangladesh, Pakistan, and India (representing the three most populous countries of the eight countries making up South Asia). The research was a pilot study to ascertain local feasibility and acceptability of a non-pharmacological intervention focussed on hearing rehabilitation in PwD [the SENSE-Cog intervention (39, 40)], adapted for the South Asian context. We report: (i) how the local research teams co-developed PPI activities with PPI stakeholders in each site; (ii) the nature of each site's PPI activities; and (iii) the impact of the work through the reported experiences of the researchers and the PPI stakeholders. The operational framework for our approach was based on the Wellcome Trust's “Public Engagement Onion” model (41) (Figure 1). We used GRIPP2 (42) (Guidance for Reporting Involvement of Patients and the Public) checklist to report our PPI activity (Supplementary File 1).

## Background to the Feasibility Study SENSE-Cog Asia

SENSE-Cog Asia is an ongoing feasibility study of a psychosocial intervention to improve quality of life in PwD through enhancing hearing function, culturally adapted for South Asian settings. Based on the European SENSE-Cog trial (40, 43) (www.sense-cog.eu), this single arm, open-label, study has four phases: (1) cultural adaptation of intervention; (2) feasibility and acceptability evaluation; (3) capacity and capability building for dementia research; and (4) PPI. Participant dyads (PwD and their care partners) are being recruited across the study sites. Each dyad receives the adapted SENSE-Cog intervention, over an 8-week period, delivered by a trained “Hearing Support Practitioner”. The intervention comprises the following components: clinical assessment and provision of hearing devices by an audiologist for the PwD; adherence support with hearing aids (or other hearing devices) for the PwD and their care partner; and knowledge, awareness and communication skills' training on sensory-cognitive impairment (including dementia education) for care partners. The main outcomes are feasibility and acceptability of the intervention. Exploratory outcomes are quality of life and other dementia-related outcomes. Below, we elaborate on Phase 4 of the project, the PPI activity.

## Methods

### Phase 4 Process: Patient and Public Involvement

#### Project Team

The PPI project team comprised two researchers (JM and SS) based in Manchester (UK) and seven researchers based in each of the South Asian settings with backgrounds in medicine, social work, occupational therapy, health sciences, speech therapy, audiology, psychology, physical therapy, and nursing. The seven researchers were identified from each clinical site and took on the role of local PPI coordinator. Site principal investigators were also involved, to oversee support for PPI coordinators in embedding PPI within the local research sites.

#### Setting

The research sites were in Pakistan (Lahore, Karachi and Rawalpindi); India (Mysuru, Chennai and Bengaluru); and Bangladesh (Dhaka). This work took place from March 2019 to March 2020.

#### Training and Support

PPI was a new concept for the designated researchers, thus, to equip them for their new role as local PPI coordinators, the UK-based researchers delivered two half-day PPI training sessions *via* video conference. We adapted the training from a previous PPI training we used in Europe (15). The training covered the principles of PPI, as well as operational aspects regarding how PPI coordinators could recruit and establish a PPI group and implement PPI activities. Site principal investigators also attended the training. The PPI training information was translated into local languages, case studies used for training were set in the local settings with South Asian names, and images used reflected the local diversity. Although we provided the basic training on the principles of PPI to raise awareness and embed the PPI concept in their work programmes, more importantly, we were guided by the local PPI coordinators who led the initiatives on addressing the cultural diversity represented within each study site.

After the PPI coordinator training, our initial plan was to have regular team meetings with the PPI coordinators *via* video conference to create a sense of doing the work together. However, local challenges due to technology provision in some sites precluded this. Instead, we conducted one-to-one meetings with PPI coordinators to support them in preparing and planning for their PPI groups and activities. One-to-one meetings included discussions about whom to involve as PPI contributors, what resources were needed to support the PPI contributors, and how the PPI coordinators role differed from that of their usual role as researchers. Local PPI coordinators were also supported in developing their knowledge, values, and attitude regarding PPI in research. Thus, the approach was to facilitate the local PPI coordinators to implement PPI practice as well as to learn simultaneously through its application in the research project.

#### Implementation Process of PPI

We use the term “public contributor” to refer to the patient and public members involved in the PPI activities in this project. Each local PPI coordinator recruited and set up their PPI group differently across sites, depending on the local context and in addressing local social and cultural issues. PPI groups were set up in each clinical site and consisted of between 2 and 4 public contributors. The numbers of PPI contributors recruited were kept to a minimum, to allow the PPI coordinators to effectively implement the learning from the training into practice and also manage and support a PPI group who were not familiar with the PPI concept. PPI contributors were recruited using flyers (Supplementary File 2) disseminated through local medical charities and patient representative organizations. In addition, PPI coordinators and representatives from local medical charities and patient representative organizations also communicated verbally about the PPI opportunities with existing patients and carer groups and other social networks to ensure the inclusion of people with illiteracy issues. Protocols to manage transport and reimbursement for PPI contributors were developed locally. Public contributors included people with dementia or memory problems, care partners, or community members with an interest in contributing to research. There were no set inclusion or exclusion criteria for recruiting PPI contributors, unlike a strict research study, the requirements were quite loose as PPI work attempts to get the widest, most inclusive and representative viewpoints and perspectives to inform the research work. Individuals required no specific skills to join the PPI groups, but needed to have lived or caring experience of dementia or a special interest in dementia.

The PPI coordinators worked with their PPI group to familiarize them to the dementia research project (5) and to acquaint group members of their role within the project. PPI contributors role was to provide advice on the running of the dementia research project (5) and/or work with the research teams to plan, make decisions and develop dissemination activities. PPI groups were provided with clear questions to aid the researchers to gain meaningful input. Meetings took place monthly or as required by each site. We used monitoring forms (Supplementary File 3) to capture the feedback from the PPI activities to demonstrate the impact of PPI (Table 1). On-going one-to-one support for PPI coordinators was provided by the UK-based PPI team (JM and SS), *via* telephone, emails, and skype meetings.

**Table 1:** Dementia-related PPI activities and impact across the seven sites based on the “Public Engagement Onion” model.

**Public engagement onion spectrum**	**Activity type**	**PPI activities**	**Impact**
Collaborating	Making decisions	PPI groups set-up	• Endorsed research relevance. • Validated intervention. • Contributed to intervention development. • Identified awareness and education needs on the research topic.
Consulting	Informing decision-making	Recruitment material and flyers, information documents for dementia research	• Documents reworded and inappropriate English words were translated.
		Combined a public engagement and awareness-raising event with a survey of professional stakeholders	• Ascertained knowledge, awareness and practice on the research topic. • Survey data guided study design and recruitment.
		Dementia care skills event for professionals	• Planned event and identified key topics. • Inter-professional collaboration and dialogues engaged professionals normally not involved in decision-making.
		Assessment of hearing screening for older adults	• Questions re-phrased to make it relevant culturally.
	Understanding thinking	Discussion group panel with professionals	• Reviewed and suggested amendments to topic guides. • Developed questions for discussion.
Informing	Stimulating thinking	Dementia awareness role-play in residential care homes	• Planned role-play and highlighted issues on carer burden issues and tell-tale signs of dementia.
		Dementia awareness event for public	• Contributed toward reducing stigma in the community.
	Information	Dementia awareness radio programme	• Reviewed topics for the radio programme. • Invited to plan for future dementia awareness sessions.
		Dementia information sheets	• Reviewed ease of understanding, readability, alternative wording and images.
		Dementia awareness community walk	• Contributed to raising of awareness.
		World Alzheimer's day poster competition	• Contribute toward reducing stigma in the community.
		Dementia newsletter	• Reviewed wording and topics relevant to public members.

We encouraged the PPI coordinators to adopt the “Public Engagement Onion” model (41) (Figure 1), to help them decide with their PPI groups on the PPI activity to be undertaken. Different sites chose different layers representing one or two of the “rings” in the “Public Engagement Onion” model (31). The “Public Engagement Onion” (41) offers a range of approaches in different forms, as there is no one optimal involvement approach (9, 15, 44, 45). The rings consist of basic engagement activities with larger audiences to more intense activities with small groups and having a greater impact.

#### Research Teams' Experiences of PPI

We conducted a short survey using self-completed questionnaires to understand the entire research teams' (*n* = 18, across all sites) experiences of applying PPI in dementia research and to add depth to the reported learning. We included researchers, research assistants, and principal investigators who were involved in supporting the PPI coordinators in implementing, supporting, and conducting PPI group meetings. The questionnaires included items relating to any previous PPI involvement experience, their view on the importance of PPI and how PPI influenced them or their work. Items included a free text box to allow the respondent to explain their answer or give further insights. The survey questionnaire is provided in Supplementary File 4. The surveys were completed anonymously. We analyzed the responses quantitatively supported by qualitative thematic analysis of open text responses.

#### Ethics Statement

We did not require ethical approval for the involvement of patients or public, as they are not acting in the same way as research participants (46) and no data were collected directly from PPI contributors. We included safeguarding aspects in the PPI coordinators training modules to ensure the protection of PPI contributors, including maintaining confidentiality, distress protocols, and correct training of coordinators. For the researcher survey, responses from the research team were collected anonymously with informed consent.

## Results

### Implementation of PPI

A key challenge for PPI coordinators was explaining the concept of PPI, which was new to potential PPI contributors. Due to the lack of awareness of the PPI concept and doubts regarding the benefits of PPI in general, some individuals were reluctant to be involved and had to be encouraged. The main concern raised was that working on an equal footing with health professionals in an advisory capacity was new territory for them, and they were uncertain about what was expected of them. They expressed doubt about whether their involvement could be beneficial or valued.

PPI coordinators were concerned about finding “suitable people” and recognized the need to approach potential individuals directly to explain the role and the concept of PPI. They spent a substantial amount of time explaining about PPI at different community centers, with community groups and at public meetings, at care homes and outpatient clinics. Other challenges reported included low general and health literacy levels, travel time, and the financial cost to attend the meetings, particularly for those traveling from rural areas. These issues were addressed by assuring individuals with low literacy that PPI coordinators would verbalize all the information using local dialects and collate the feedback by taking notes. Inclusion of less educated participants, or those from rural areas, was considered important for inclusion. For those traveling from rural areas, the PPI meetings were arranged around hospital appointments to save travel time and avoid additional costs. In addition, reimbursement for the travel cost and time were provided, however some sites decided to only cover the travel expenses. PPI coordinators reported that it was unusual to pay public members expenses and reimbursements for meetings. This added another layer of complexity for most sites as there were some infrastructure challenges identified by PPI coordinators. There was an absence of a policy or guidance for remunerating public contributors in research projects and a lack of guidance on what was reasonable compensation. Therefore, the team developed expense sheets for the project and agreed on local rates for reimbursement that were realistic but still guided by Public Involvement Standards guidance (47), which were established in the UK for UK-based PPI. Researchers questioned whether this aspect of the guidance was contextually appropriate.

A total of 27 people were recruited for the PPI activities across the seven sites, consisting of 8 PwD, 14 carers and 5 members of the public. The PPI groups in each site decided on the preferred meeting times and settings. The first meetings with the PPI groups were mainly focussed on equipping them to acknowledge that their knowledge and expertise of the lived experiences were as important as clinicians and researchers, although it was different.

### Impact of PPI on the Research Project

The impact of PPI on a given research project can be characterized by changes to the research, as well as researchers and PPI contributors and the wider community (48). Tables 1, 2 illustrates the demonstrable impact of the different PPI activities on different aspects of the research project.

**Table 2:** Case studies of two PPI groups and the impact of their PPI activities.

**Case Study 1: Mysuru, India site**
The Mysuru PPI advisory group advised on the development of a dementia awareness-training event for members of the public during Alzheimer's Awareness month in September 2019.
The Mysuru PPI advisory group attended a series of 2-hour meetings. The group consisted of carers and volunteers. The meeting was held on days agreed amongst the group that would be the most convenient.
The aim of the meeting was to gather views on dementia awareness training at a very early stage of the planning. Three contributors participated in the meeting to help develop the dementia awareness training and one member provided input remotely *via* telephone. Face to face, group meetings enabled the PPI contributors to discuss their views and providing the opportunity to all the contributors to put forward their ideas. The PPI group provided feedback about each of the three components: • Where and how the training should be advertised. • The key topics to be covered by the training. • The agenda
The research team and the PPI coordinator worked with the PPI group to develop plans for awareness training. They focused on whether the language used to introduce the subject of dementia in the training was appropriate. There was agreement that the word “dementia” raises concerns. So the suggestion was that the word “memory loss” should be used and as more sympathetically worded, and embraced varying features of the condition. The group also highlighted the need for more information about local support available to persons with dementia, and the need for awareness materials to be provided in Kannada (local language). The feedback from the PPI contributors resulted in the following changes: • The training focussed on preventative measures and signposting resources for care partners. • Training included activities to keep the person with dementia busy and engaged. • The training included lists of do's and don'ts for care partners. • Training included prompts for care partners to discuss that “it is Ok to have a nurse to help” and issues on stigma on receiving care from nurses, to overcome high levels of stigma and discrimination in the community. • Training content used layman's terms as suggested by PPI contributors e.g., to use the terms “memory loss” instead of dementia.
**Case Study 2: Bengaluru, India site**
The Bengaluru site consisted of a “virtual PPI advisory group” with eight public contributors. PPI activities were organized *via* a virtual discussion group. To ensure equality of involvement, those who could not attend the virtual sessions or did not have services to participate, PPI coordinators arranged a consultation with them using telephone or face-to-face meeting dependent on the PPI contributor's preference. The approach taken by the Bengaluru PPI coordinator was firstly to set up a series of discussion topics for the groups to discuss issues and challenges faced by PwD and care partners, to set the context. Thereafter, discussions led to the introduction of the dementia study and the relevance of PPI input. Consecutive discussions consisted of items about the study intervention design, Hearing Support Practitioner's communications manual and patient information sheet to receiving feedback that could help the research team improve its documentation for the trial. • Feedback highlighted that care partners, due to various reasons, sometimes limit the participation of the PwD but the Hearing Support Practitioner's should ensure that the PwD is encouraged to talk during sessions. • Suggestion for re-wording recommended for the participant information sheet and informed consent sheet, emphasizing that both the PwD and care partners are warned about what the research study entails, and commitments involved. • The relevance of the intervention was recognized and approved by the group.
The group recommended the benefits of wearing the hearing aid and how it could reduce the burden on care partners to be shared with everyone, and not just as part of the research activity. The issues relating to equality between researchers and study participants highlighted as important factors by ensuring accessibility for all, and not the selected few.
The feedback from the PPI advisory groups resulted in the following changes: • Hearing Support Practitioner's communications manual reworded and notes included to ensure that the participation of the PwD is encouraged during the intervention, which could be limited sometimes by care partners. • Patient information sheet and consent forms reworded using the simple local language. • Researchers assured through PPI advisory groups validation that PwD and care partners would benefit from the intervention. • Researcher's notes as part of the intervention delivery emphasized the need to explain to the care partners and patients about their role in dementia research, to ensure equality by promoting the study widely to provide the opportunity for all to participate in the research study. • Bengaluru research site promoted volunteers led outreach activities for promoting the rights and dignity of people living with dementia and promoting the benefits of using a hearing aid for PwD.
Other issues identified by the group highlighted: • The need for general awareness and education about the research topic. • Researchers to explore options of providing virtual session as part of the intervention.
The research team addressed the transport-related issues for PPI advisory group members by the use of smartphones in urban and suburban settings with a view to enabling many care partners to participate with the online group without affecting the caregiving responsibilities and increasing digital literacy.

#### Range of PPI Activities

As shown in Table 1, each site chose a different type of PPI activity within the “Public Engagement Onion” model, ranging in level and magnitude of involvement through to engagement. For example, the Bengaluru site undertook PPI-led modification of the study participant information documents, including patient information sheets, for use in all sites. Groups in Lahore, Karachi and Rawalpindi undertook various public engagement events, ranging from dementia awareness role-play in residential care homes, discussion groups, radio program and developed dementia awareness newsletter (Supplementary File 5). The group in Mysuru consulted on the study protocol and conduct, providing insight into local adaptation and co-developed dementia training for professionals and dementia awareness-raising training for public members. Chennai's group developed dementia information sheets for use in the intervention trial, after ethical approval. In Dhaka, the group supported audiologists in adapting a hearing screening checklist for older adults, which were needed for the study protocol. Finally, in Dhaka, the group chose to conduct a large-scale public engagement event to raise awareness of sensory-cognitive health, attended by 100 participants. The event provided a platform for key stakeholders (public, doctors, medical students, occupational therapists, social workers, and physiotherapists) to have a dialogue about dementia and hearing impairment that had not previously been discussed in such settings. Key discussions were focussed on strengthening the legal laws on the Mental Health Act in relation to people with dementia and lacking the capacity to consent and focus on strengthening a workforce to address dementia care through capacity building. During the event, 56 participants filled out a Knowledge, Attitude, Practice [KAP (49)] survey regarding sensory-cognitive health (the results of the Dhaka PPI engagement event survey are reported below). KAP is used to gather data on what is known, assumed and practiced in relation to a specific topic (49).

Each site in this study is a case study in their own right. However, we have chosen to use two case studies in this paper to reflect the most interesting in terms of the approach used and the PPI tasks undertaken by two PPI groups in India. We illustrate in Table 2 how PPI was implemented and the impact of their activities.

#### Research Teams' Awareness of PPI Survey Results

Ten researchers took part in the survey. Of these, five were PPI coordinators (researcher background), one researcher, two research assistants, and two principal investigators. Most researcher respondents (80%) reported that they had no previous experience of PPI work; 10% was unsure and 10% reported having some experience of PPI, mainly with professional stakeholders. All participants viewed PPI as an important factor in dementia research. Most of the participants said they are “very likely” (70%) or “likely” (20%) to apply PPI principles to other projects or as part of their work in their department, whereas, 10% were neutral on this subject. The positive responses in applying PPI in their work were supported by comments outlined in Table 3.

**Table 3:** Exemplar quotes illustrating the impact of the PPI activities on researchers.

***Theme***	***Quotes***
Unique experience of illness and insights into the needs and priorities of a PwD and care partners	“*Involvement in this project help me to interact more with the patients this was the experience in understanding the family better” (ID9)* “*Working with the public contributors made it a different experience, it gave us insights into what are the real challenges for people like the cost of traveling into the hospitals, particularly from the villages, it*'*s far and for some it means having to arrange overnight accommodation, which is additional cost for them. So it*'*s useful to get that insight.” (ID8)* “*PPI is very helpful to getting understand your targeted population and their problems when you involve them in group discussions.” (ID4)* “*Throughout the Sense Cog Asia project I have learned a lot. It was one of the best opportunity to learn purely about the challenges of patients and their care partners. Involvement of public in this project was quite challenging task. True representation and involvement of public in project is only can achieved if they are given opportunity to give us their valuable opinion about project design and implementation.” (ID1)* “*Gives insight into people*'*s lives and how they are dealing with the conditions, it helps us as researchers to understand some of the changes they would face in relation to dementia research and what the intervention means to them” (ID7)*
Improved understanding of PPI	“*Co-creation helps us to understand ground reality. It also helps to frame need based research problems.” (ID5)* “*You get to now (sic) how you can you improve research design and strategies.” (ID4)* “*Yes. PPI learn me research differently.” (ID9)* “*Because it improves the relevance of the study and more practical goals or research questions can be taken up” (ID3)* “*Yes, definitely I will do that because this is the best way to evaluate your project connection with your participants moreover it helps in any trial to investigate the progress of your project outcome.” (ID1)*
Challenges of PPI	“*Time consuming. Conflict of opinion and level of interest of public makes it difficult to achieve the outcome of this project” (ID1)* “*It's new idea. I need to give more time in research project” (ID2)* “*Patients getting more involved and trying to rule over the researcher. Coordinating timing for meeting” (ID3)* “*Most of the carers are daughters-in-law and I think they are hindered from talking openly about certain things because if they were to do it, it is seen as disrespecting the elder member. May be we need to think about it for the intervention too; that might be an issue” (ID8)* “*It's time consuming, I think because it's new thing for us, it has taken us longer to understand it fully and also to adapt to a new way of working” (ID7)*

**Figure 2:**
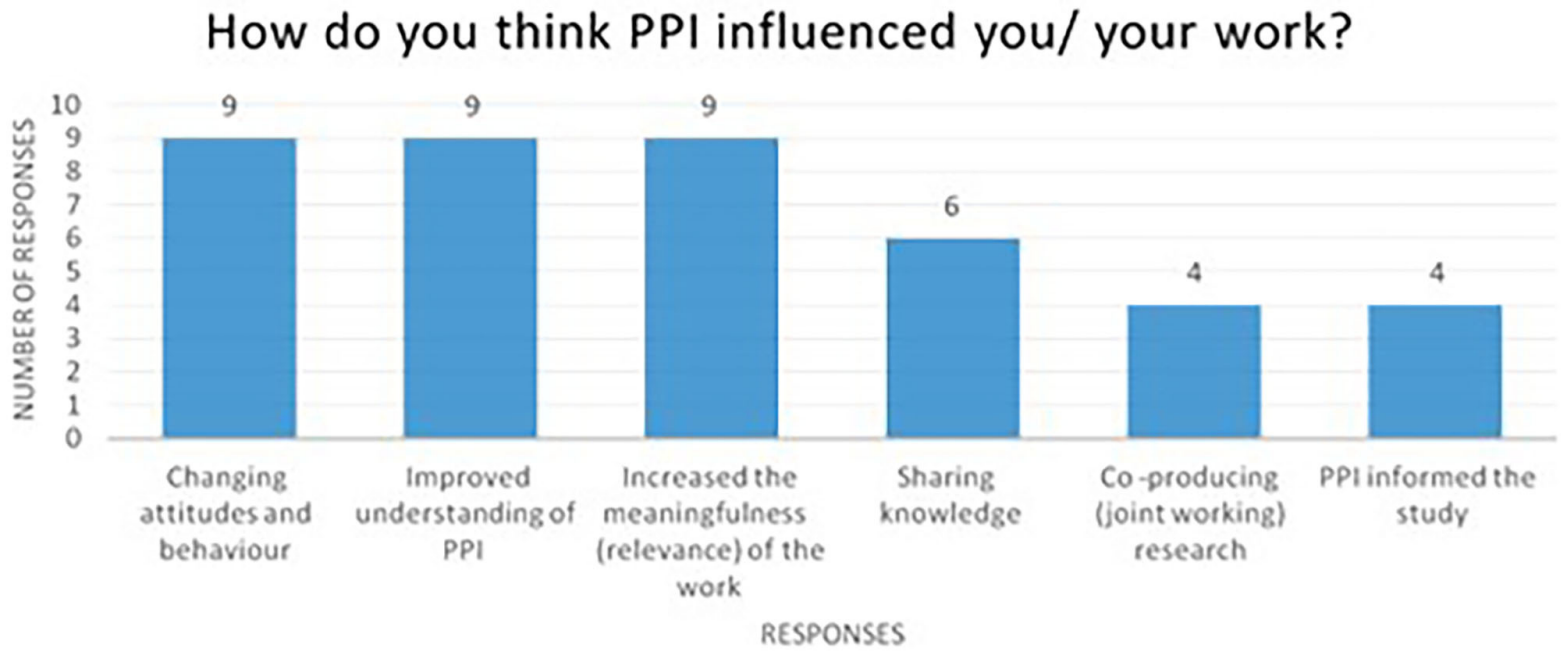
Survey results showing how PPI influenced the researchers and/or their work.

As shown in Figure 2, 90% of the researcher respondents reported that they had improved their understanding of PPI and that PPI had increased the meaningfulness and relevance of their work. Sixty percent said that PPI enabled knowledge sharing and 40% felt that PPI informed the study and led to joint working on the research project.

Finally, 90% of respondents reported that PPI changed their attitudes and behavior, including a broadening of their perspectives about the challenges faced by patient and care partners, and understanding the types of challenges that might be faced by PwD and care partners in taking part in the intervention. It also provided an opportunity for researchers to interact with PwD and their care partners outside of the research study. Some researchers reported how the PPI activities had helped them to understand what needs to be done from an organizations perspective in helping to raise awareness about dementia for the public. Exemplar quotes illustrating the impact of the PPI activities on researchers are shown in Table 3.

The disadvantages of PPI were also reported by respondents. These included the view that PPI was time-consuming and was an added burden to the already busy role of a researcher. They viewed PPI as a new way of working, which took time to fully understand and adapt to. A few respondents commented how PPI presented conflicts of interest from public contributors and made it difficult to achieve the outcome of the study, particularly as it challenged the authority of the researcher. For example, one participant commented “*Patients getting more involved and trying to rule over the researcher”* (ID3). Another researcher highlighted a cultural challenge for PPI contributors as family care partners, particularly for daughters-in-law. The researcher felt that in-group settings, particularly in settings where women are not often asked for their opinions, the adult children, or daughters-in-law were hindered from talking openly about issues relating to care partner burden, as it was viewed as “*disrespecting” (ID8)* the older adult they are caring for. Another point highlighted by a researcher was the difficulty of persuading those with “*cognitive deficiency” (ID10)* to attend the meetings and the challenges arising from involving them in the meetings.

#### Dhaka PPI Engagement Event Survey

Fifty-six participants completed the KAP surveys in Dhaka and were included in the analysis. Findings are outlined in Tables 4, 5.

**Table 4:** Dhaka KAP survey demographics.

	**Number**	**Percentage**
**Gender (*****N*** **=** **55)**		
Female	21	38.2%
male	34	61.8%
**Years in profession (*****N*** **=** **55)**		
<2	5	9.1%
2–5	11	20.0%
5–10	18	32.7%
>10	21	38.2%
**Highest qualification (*****N*** **=** **55)**		
Postgrad	19	34.5%
Degree or equivalent	22	40.0%
Dip or equivalent	10	18.2%
A level or equivalent	2	3.6%
GCSE or equivalent	1	1.8%
Other qualifications	1	1.8%
**Job title (*****N*** **=** **55)**		
Service manager	10	18.2%
Registered nurse etc.,	1	1.8%
Care worker	6	10.9%
Allied health	34	61.8%
Other	6	7.3%
**Received training in dementia (*****N*** **=** **56)**		
No	51	91.1%
Yes	5	8.9%

**Table 5:** Dhaka KAP survey responses.

**Knowledge items**											
**Questions**	**Strongly disagree**		**Disagree**		**Neutral**		**Agree**		**Strongly agree**		**Total**
	***N***	**%**	***N***	**%**	***N***	**%**	***N***	**%**	***N***	**%**	***N***
I am aware of brief hearing tests that could be used with PwD	13	23.2%	5	8.9%	7	12.5%	26	46.4%	5	8.9%	56
I have the training and expertise to administer and interpret the results of a brief hearing test	16	29.1%	13	23.6%	8	14.5%	15	27.3%	3	5.5%	55
I am aware of, and would be able to use, appropriate referral pathways for PwD who failed a brief hearing screen	12	21.4%	7	12.5%	9	16.1%	22	39.3%	6	10.7%	56
I am confident in helping PwD with use of assistive hearing devices	11	20.0%	3	5.5%	19	34.5%	17	30.9%	5	9.1%	55
**Attitude toward hearing support for PwD**											
**Questions**	**Strongly disagree**		**Disagree**		**Neutral**		**Agree**		**Strongly agree**		**Total**
	***N***	**%**	***N***	**%**	***N***	**%**	***N***	**%**	***N***	**%**	***N***
A brief hearing screen would be acceptable to PwD	1	1.8%	10	18.2%	6	10.9%	23	41.8%	15	27.3%	55
I would find clinical guidelines for assessing and managing hearing impairment in PwD care useful	4	7.3%	5	9.1%	6	10.9%	23	41.8%	17	30.9%	55
Most PwD who need a hearing aid (or other assistive hearing device) use one effectively	11	19.6%	12	21.4%	9	16.1%	17	30.4%	7	12.5%	56
**Practice related to hearing support for PwD**											
**Questions**	**No**				**Yes**					**Total (*****N*****)**	
	***N***		**%**		***N***		**%**				
Do you carry out testing or checking of hearing aids?	36		65.5%		19		34.5%			55	
Does your facility have specifically designated staff that are responsible for the care of hearing impairments (e.g., putting a hearing aid in, changing batteries)?	44		83.0%		9		17.0%			53	
I have training and support to use hearing aids, amplifiers etc.,	50		92.6%		4		7.4%			54	

##### Demographics

Respondents were generally male (61.8%) and equally, a majority (61.8%) of respondents were allied health professional workers. Respondents were relatively experienced (32.7% of respondents having worked 5–10 years and 38.2% with over 10 years' experience) and moderately highly qualified. However, only 8.9% reported having received training in dementia awareness.

Knowledge: Over 50% of respondents reported being aware of brief hearing screening tests, but don't have the training and expertise to administer and interpret the results and 50% reported being aware of referral pathways. Although, 40% of respondents reported being confident in helping PwD with use of assistive hearing, other respondents reporting neutral and lower, cited the main reason given for not being confident was lack of training (Supplementary File 6).

Attitudes: Most respondents (69.1%) agreed that hearing screening would be acceptable to PwD and 72.7% agreed they would find clinical guidelines for assessment and management of hearing useful. Additionally, respondents (41%) reported that most residents who needed to use a hearing aid did not use them effectively. The most reported reasons for ineffective use were aids not effective, not being tolerated, aids hard to use or not fitting correctly.

Practice: Most respondents (65.5%) reported they did not carry our testing or checking of hearing aids. Only 17.0% of respondents reported that there were specially designated staff responsible for hearing care in their facility. Most respondents (92.6%) reported they did have any training and support to use sensory support equipment.

## Discussion

Through this project, we established a community of researchers and public contributors undertaking PPI practice by encouraging researchers to consider and explore PPI methods, develop a positive attitude toward PPI, and implement PPI. The purpose of capacity building was to facilitate a bottom-up approach, consistent with the ethos of PPI, of gaining self-confidence, and learning about PPI in research. To our knowledge, this is the first PPI for research involving PwD, persons with memory problems, care partners, and community members in LMICs.

We purposely used a multifaceted framework of implementation and evaluation, based on the “Public Engagement Onion” (41). A bespoke approach, involving different underlying theoretical approaches, is appropriate, considering the diverse nature of the different study sites, which differed by country, language, research experience and dementia awareness and expertise, and the novelty of the concept for both researchers and public contributors. Avoidance of “off-the-shelf” approaches has been supported by some PPI authors [i.e., Greenhalgh et al. (22)] and this ensured that the PPI stakeholders at each site could select the approach most suited to them, aligned with a plurality of theoretical frameworks, and recognizing the importance of context, making it relevant to the people “on the ground.” Introducing an innovation model such as PPI does not necessarily transform to uptake in all settings, as consideration needs to be given to the local socio-cultural and health system contexts when implementing PPI. It should involve working and engaging with local communities, stakeholders and community leadership networks, and viewed as a continuous learning process.

Our work included a focus on (1) study feasibility and preparation; (2) partnership building for dementia research capacity and capability building; (3) education and awareness-raising; and (4) ensuring power balance and equity in researcher-participant relationships. Regarding the latter, as dementia research develops in LMICs, particularly in collaboration with international partners, it is critical, at the outset, to address issues of equity and inclusion (50) and it is important to assume and be vigilant for the risk of research imperialism for any international collaboration, and PPI may have an important role in addressing this. PPI can support this by placing the voices of PwD, persons with memory problems, care partners, and community members (28, 51) at the center of the research, thus de-centralizing the researcher, who is often a medical professional representing a power imbalance (52) with PwD and their care partners (53–56). Illustrating this emancipatory aspect of PPI, our researcher survey highlighted the challenges of public contributors “taking control” or being treated as equals in the partnership. This represents “uncharted waters” for many medical professions in LMIC settings like South Asia (57, 58), where the relationship between medical professionals and patients is more vertical compared to many HICs (59), and communication is determined by accepted social differences (52, 60). However, it is worth noting that hierarchical dynamics are still common in some cultural contexts, holding an authoritative hierarchical position within their communities, and power imbalances naturally emerge between researchers and subjects, and not necessarily due to research imperialism and colonialism, but due to local sociocultural practices. In this context, PPI has the potential to foster equity by disrupting traditional boundaries of social structure, which is central to a partnership approach.

Many PwD in LMICs fall into the “triple jeopardy” of being older, female, and from the poorest communities. This can be conceptualized through the lens of intersectionality (50, 61), where several socially determined risk factors coalesce to predispose and precipitate the emergence of dementia and perpetuate poor outcomes (62). Thus, when embarking on research with this vulnerable population, the needs and perspectives of the PwD and their family must be prioritized. A robust PPI approach will support this and address the social justice principle of “inclusion.”

The political imperative, which holds that knowledge should be co-created by researchers and lay stakeholders, was demonstrated in several of our PPI activities, although it emerged as a challenge not endorsed as an outcome by some researchers in the survey. For the researchers who reported that the PPI work *had* helped them focus their research on the needs of the public contributors, this represented a significant paradigm shift away from paternalism toward partnership.

A significant practical barrier during PPI implementation work was the recognition of how payment to PPI contributors, an important element of INVOLVE guidance (63), might not be appropriate nor is practiced in most LMIC settings. This compounds issues of exclusion, in that those who get involved are those who can afford the time and money to do so. The Bengaluru case study illustrates how the use of virtual groups can enhance PPI access for a particular group; however, this approach also has its limitations by excluding those without access to technology. Therefore, during PPI implementation, researchers should consider how to reach beyond clinical and hospital settings and “go to the people.”

Since a focus in underserved areas is often on the most impactful research in the shortest timespan to address areas of greatest need, studies of interventions with an existing evidence-base are often undertaken. An example of this is the global adaptation and implementation trial of Cognitive Stimulation Therapy for dementia (38, 64). Adopting an intervention developed elsewhere into a context with a markedly different language, socioeconomic and cultural context requires adaptation prior to evaluation to enhance the appropriateness, uptake and chance for subsequent “scale-up” of the intervention (6, 46, 65–67).

Although PPI in dementia research has progressed substantially, evidence supporting the impact of PPI is still developing and descriptions of impact are sparse and frequently lack consistency due to inadequate conceptualization, and inconsistent reporting (16, 44, 68–72), thus rendering the evaluation of impact difficult. Nonetheless, it is critical to develop a strong evidence base for PPI by demonstrating impact (17, 73), as we have illustrated here (Tables 1, 2, researcher survey, monitoring forms), and thus moving the PPI agenda forward in a significant and applicable way (17, 22, 73, 74).

### Limitations

We recognize that the PPI concept and the approach we introduced into SENSE-Cog Asia study could be viewed as Eurocentric and to a certain extent as a form of colonialism by some researchers in LMIC. However, the core of our work on PPI is about the democratization of health and health knowledge, which in many LMIC (and HIC, particularly non-English HIC) settings is challenging as structures remain moderately vertical and patriarchal. The model of “doctor knows best” is still very prevalent. Thus, PPI could have a “disruptive” role in breaking those traditional boundaries in LMIC settings, which may be positive.

Principles of PPI training and support include (17, 47, 73) training public contributors regarding the basics of research. Although we explained the PPI contributor's role in providing input to support research, we provided only basic research training to PPI contributors due to limited resources and time constraints. Much of our time was spent on introducing the concept of PPI. Moreover, PPI coordinators were identified locally within the research team, which may have contributed to power imbalance (75), whereas independent PPI coordinators may have been more appropriate.

In addition, due to limited resources, we did not include the perspectives of PPI contributors to capture their view on how they embraced the PPI concept, their experience and its impact, which is a vital perspective to capture when implementing new concepts. These data would have been valuable by enabling us to explore alignment between the perceived experiences of PPI coordinators and contributors. The absence of these data may make the “Implementation of PPI” section in the results section appear somewhat subjective. However, this subjective aspect is also important, as it represents as proof of concept to establish feasibility and acceptability of our approach, which was the main aim. Future work in this area can apply more rigor to the methodology and training and increase the rollout to support other studies and ensure the sustainability of the PPI groups.

Despite our efforts to be inclusive, PPI contributors recruited in this study were to a certain extent biased toward a particular demographic of educated, literature and largely middle-class people. For future research studies in newly developing research centers, such as those sites with whom we worked with need to be mindful in addressing the issues of inclusion in PPI recruitment, by increasing the reach to rural areas, poor and vulnerable, and digitally disadvantaged communities.

## Conclusion

We have synthesized our learning and reflection on PPI capacity building in LMICs in some South Asian contexts and emphasized the need to strengthen PPI practice in LMICs, particularly for newly emerging areas of research and practice, such as dementia care. PPI must be recognized as an integral part of applied research in LMIC countries, but requires sufficient investment in time, resources, and commitment to ensure PPI is effectively led and research outcomes are relevant to the intended beneficiaries. “Learning by doing” (45, 74, 76) will be necessary to make more explicit the various factors that support and inhibit PPI processes and tailor different types of involvement practice (22) to change the research landscape globally.

## Data Availability Statement

The original contributions presented in the study are included in the article/Supplementary Material, further inquiries can be directed to the corresponding author/s.

## Ethics Statement

Ethical review and approval was not required for the study on human participants in accordance with the local legislation and institutional requirements. The participants provided their written informed consent to participate in this study.

## Author Contributions

JM, SSh, and IL conceptualized and led the project. JM and IL led the manuscript preparation. All authors participated in refining and implementing the work and contributed to the critical review and final version of the manuscript.

## Conflict of Interest

The authors declare that the research was conducted in the absence of any commercial or financial relationships that could be construed as a potential conflict of interest.

## Abbreviations

PPI: Patient and public Involvement
PwD: Person with Dementia
LMIC: Low- and middle-income countries
KAP, Knowledge, Attitude: Practice.
