# Supplementary material for: Patient and Public Involvement for Dementia Research in Low- and Middle-Income Countries: Developing Capacity and Capability in South Asia
Source: Front Neurol. 2021 Mar 23;12:637000. doi: 10.3389/fneur.2021.637000 (PMC8021770; doi:10.3389/fneur.2021.637000)
Supplement: Supplementary file 1 [file Data_Sheet_1.zip › Supplementary File 1.docx]

**Supplementary file 1 – GRIPP2 short form**

| Section and topic | Item | Reported on page No |
| --- | --- | --- |
| 1: Aim | Report the aim of PPI in the study | 4 |
| 2: Methods | Provide a clear description of the methods used for PPI in the study | 5,6 |
| 3: Study results | Outcomes—Report the results of PPI in the study, including both positive and negative outcomes | 7,8,9 |
| 4: Discussion and conclusions | Outcomes—Comment on the extent to which PPI influenced the study overall. Describe positive and negative effects | 10,11,12 |
| 5: Reflections/critical perspective | Comment critically on the study, reflecting on the things that went well and those that did not, so others can learn from this experience | 11,12 |
