# Supplementary material for: Patient and Public Involvement for Dementia Research in Low- and Middle-Income Countries: Developing Capacity and Capability in South Asia
Source: Front Neurol. 2021 Mar 23;12:637000. doi: 10.3389/fneur.2021.637000 (PMC8021770; doi:10.3389/fneur.2021.637000)
Supplement: Supplementary file 1 [file Data_Sheet_1.zip › Supplementary File 2.docx]

**Supplementary file 2 - Draft Recruitment flyer guide**

**
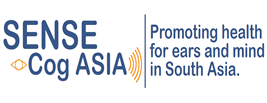
**

Organisation logo

**Would you like to help shape research aiming to support older people with hearing and memory problems?**


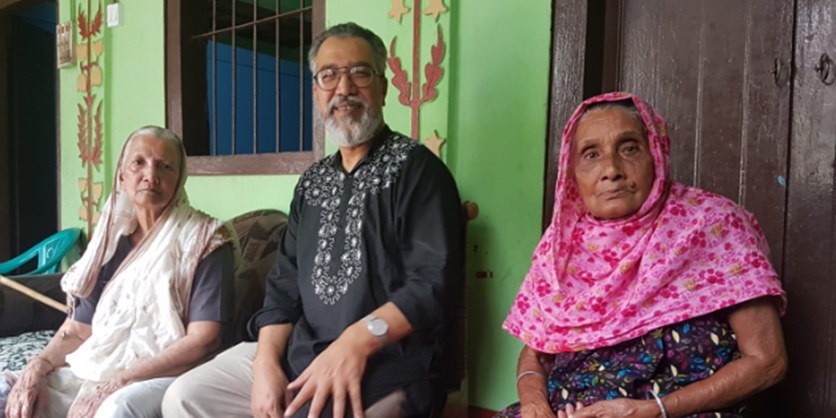


**Are you:**

- Living with memory problems and/or hearing problems?
- Caring for someone who is living with memory problems and/or hearing problems?
- From a voluntary/ community organisation that represents people with memory problems and/or hearing problems?
- Do you have a special interest in this area of work?

If yes, we are keen to hear from you to help us with our research project ‘Asian Supportive Hearing Intervention for Dementia’ (ASHID). The study is a 6 months project and will test the suitability and appropriateness of home-based intervention designed to identify and support people with memory problems (dementia) with hearing problems.

**What does this mean for you?**

• Be part of a patient and public involvement group to help us shape the research project.

• Act as a critical friend in our research. Advise and give feedback about our research.

• Attend meetings suitable for you.

• We will reimburse your travel expenses and for your time (Organisation to specify)

**Interested?**

Please contact the Patient and Public Involvement co-ordinator Name (contact details)
