# Supplementary material for: Patient and Public Involvement for Dementia Research in Low- and Middle-Income Countries: Developing Capacity and Capability in South Asia
Source: Front Neurol. 2021 Mar 23;12:637000. doi: 10.3389/fneur.2021.637000 (PMC8021770; doi:10.3389/fneur.2021.637000)
Supplement: Supplementary file 1 [file Data_Sheet_1.zip › Supplementary File 3.docx]

**Supplementary file 3 – PPI monitoring forms**

| **PPI group Site** |  |
| --- | --- |
| Research Programme | SENSE-Cog Asia - ASHID intervention |
| Project lead/ Researcher |  |
| Dates of PPI activity |  |
| PPI Group task outline | RUG members to comment on the ‘Asian Supportive Hearing Intervention for Dementia’ (**ASHID**). |
| PPI Input | Exemplar questions for PPI groups:  **Research Design**   - Did you understand what the project was about from participant information sheet? - Do you think patients will agree to participate? Is the interval between proposed study visits appropriate? - Is the researcher asking too much of the patient at each study visit? - Are the proposed visits too long/intensive? - What could be done to make the project more acceptable to patients/participants? - Other suggestions |
| PPI group feedback |  |
| Action/s taken as a result of PPI feedback:  * note actions/ changes decided by the research team based on the PPI group feedback |  |
| Action not taken with a reason * with reasons why action not taken/not possible |  |
| Any other comments |  |
