# Supplementary material for: Patient and Public Involvement for Dementia Research in Low- and Middle-Income Countries: Developing Capacity and Capability in South Asia
Source: Front Neurol. 2021 Mar 23;12:637000. doi: 10.3389/fneur.2021.637000 (PMC8021770; doi:10.3389/fneur.2021.637000)
Supplement: Supplementary file 1 [file Data_Sheet_1.zip › Supplementary File 4.docx]

**Supplementary file 4 – SENSE-Cog Asia – Researcher Questionnaire: Impacts of patient and public involvement on dementia research**

1. What is your role within SENSE-Cog Asia?

🞏 PPI coordinator

🞏 Researcher

🞏 Research assistant

🞏 Principal investigator

🞏 Other, please specify

2. Which clinical site are you in?

🞏 Dhaka

🞏 Bengaluru

🞏 Mysuru

🞏 Chennai

🞏 Karachi

🞏 Lahore

🞏 Rawalpindi

3. Have you been involved in any previous work involving patients and public members in advisory role (not research participation)?

🞏 Yes

🞏 No

🞏 Not Sure

4. Do you think PPI involvement is important in dementia research?

🞏 Yes

🞏 No

🞏 Not Sure

5. How do you think PPI influenced you/ your work? Tick all that apply*

🞏 Changing attitudes and behaviour

🞏 Improved understanding of PPI

🞏 Increased the meaningfulness (relevance) of the work

🞏 Sharing knowledge

🞏 Co-producing (joint working) research

🞏 PPI informed the study

🞏 None

🞏 Other, please specify

6. Will you apply PPI and its principles to other projects or work in your department?

🞏 Very Likely

🞏 Likely

🞏 Neutral

🞏 Unlikely

🞏 Very Unlikely

7. If yes, why?; If no, why not?

🞏 Yes (please explain)

🞏 No (please explain)

8. What are some of the advantages you have seen in involving PPI?

9. What are some of the disadvantages you have seen in involving PPI?

10. Any other comments?

Thank you for completing the form.
