# Supplementary material for: Patient and Public Involvement for Dementia Research in Low- and Middle-Income Countries: Developing Capacity and Capability in South Asia
Source: Front Neurol. 2021 Mar 23;12:637000. doi: 10.3389/fneur.2021.637000 (PMC8021770; doi:10.3389/fneur.2021.637000)
Supplement: Supplementary file 1 [file Data_Sheet_1.zip › Supplementary File 5.pdf]

Pakistan Institute of Living and Learning

Issue # 8

Neurocognitive Disorder — Dementia

Neurocognitive disorder is a general term that describes decreased mental function due to a medical disease other than a psychiatric illness. **Dementia** is an overall term used to describe symptoms that impact memory, performance of daily activities, and communication abilities.

Possible Symptoms of Dementia

- Recent memory loss: a sign of this might be asking the same question repeatedly.
- Difficulty completing familiar tasks: for example, making a drink or cooking a meal.
- Problems communicating: difficulty with language; forgetting simple words.
- Disorientation: getting lost on a previously familiar street, market or time oriented
- Problems with abstract thinking: for instance, dealing with money.
- Misplacing things: forgetting the location of everyday items such as keys, or wallets.
- Mood changes: sudden and unexplained changes in outlook or disposition.
- Personality changes: perhaps becoming irritable, agitated, suspicious or fearful.
- Loss of initiative: showing less interest in starting something or going somewhere.

Causes of Dementia		
Brain Cell Death	Alzheimer Disease	Head Injury
A Stroke	Brain Tumor	Vitamin deficiencies

Diagnosis

Diagnosing dementia and its type can be challenging. No single test can diagnose dementia, so doctors are likely to run a number of tests that can help pinpoint the problem.

Treatment: Most types of dementia can't be cured, but there are ways to manage symptoms through medication and therapies.

Self Help Message: Providing care for someone with dementia is physically and emotionally demanding.

Here are some suggestions you can try to help yourself cope with the situation:

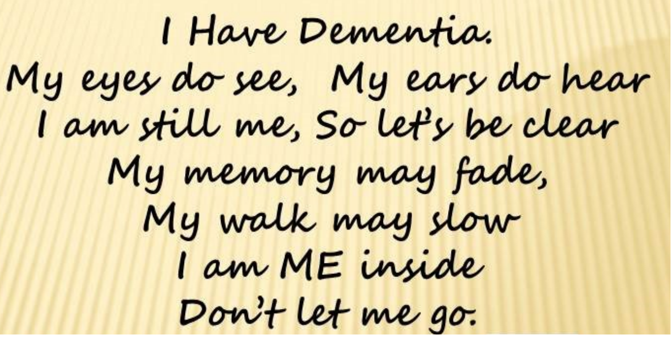

- Learn as much as you can about memory loss, dementia and Alzheimer's disease.
- Spend time with friends and family. Write about your feelings in a diary.
- Get individual or family counseling.
- Stay active and involved, volunteer, exercise, and participate in activities for people with memory loss.
- Find new ways to express yourself, such as through painting, singing or writing.
- Ask friends or other family members for help
- Take care of your physical, emotional and spiritual health

***Incase of emergency, take the patient to the nearest hospital*.**

Pakistan Institute of Living and Learning

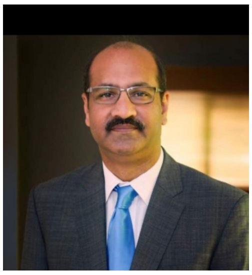

Prof Asad Tamizuddin Nizami

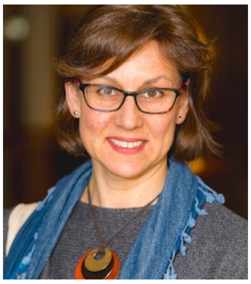

Prof Iracema Leroi

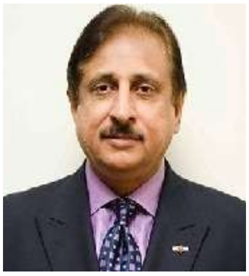

Dr. Mowadat Rana

Dementia is demeaning for humans. Memory in all its glory not only defines humans, it keeps them connected to their past, loved ones, their race, and above all their history. Its crucial for clinicians to separate dementia as a disease from cognitive decline consequent to ageing. Even more important is to identify treatable causes of dementia and also respond scientifically to those who suffer from the question "do I have Alzheimer?" Yet again it is PILL that holds promise to come to our rescue by chalking out a roadmap for dementia services in this part of the world. Thank you PILL

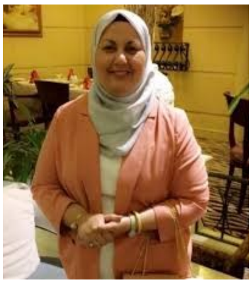

Ms. Shela Minhas

"Care for those who once cared for us"

Look, son Usman I have noticed that from few days I am forgetting things. You know that I have the habit of reading book but from previous two days I am having problem in finding my book back in morning. I am having hesitation in meeting people, the friends that meet me regularly I am forgetting their names as well and when I talk to them than I forget that what I was talking about.

Baba there is no issue anyways you are getting old now a days. As you are 65 years old now. After sometime Usman realized that his father is actually forgetting thing as he is trying to find a book in his room but which book he needs to find, he does not remember that. Then one day Usman shared all these things with his friend, who told him that, it's a disease in which a person memory and other abilities like making decision, work related to mind, interaction with people got effected. Doctors call it "Cognitive impairment or Dementia" after looking at symptoms and detailed examination of patients. After that Usman takes his father to a neurologist. Doctor told Usman after doing detailed examination and tests that his father has developed Dementia. He mentioned that it cannot be cured completely but the problems arising due to it can be reduced.

The very next day Usman took his father to a psychologist. He gave them some instructions like, the things that are under his use should not be changed, like room setting. He also said to purchase a diary for him and the regular tasks should be mentioned in it, and involved him in conversation. Then Usman followed the advice of a psychologist and all these things helped Usman's father in dealing with issues that was arising due to memory impairment.

For Care Givers

Dementia symptoms and behavior problems will progress over time. Following suggestions might help caregivers

- ***Modifying the environment***
- ***Establish a night time ritual***
- ***Occupational Therapy***
- ***Keep a calendar***
- ***Engage in Activity***
- ***Enhance Communication***
- ***Encourage Exercise***

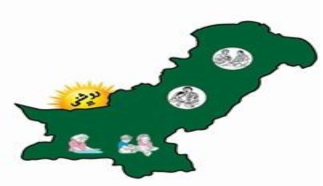

1. Montessori Intervention for individuals with memory impairment: A Feasibility study - MIRACLE

PILL has recently completed a feasibility trial on older adults with memory impairment. In this study Montessori intervention was delivered. The Montessori method is a system of teaching based on principles to promote learning and independence in children. Same approach was used for older adults and it was found to be moderately effective in improving certain behavioral outcomes such as agitation. Results of our study shows participant's positive engagement with this intervention and good retention rate. This suggests that it may be a promising form of support for people with dementia and need to be refined and develop for larger scale.

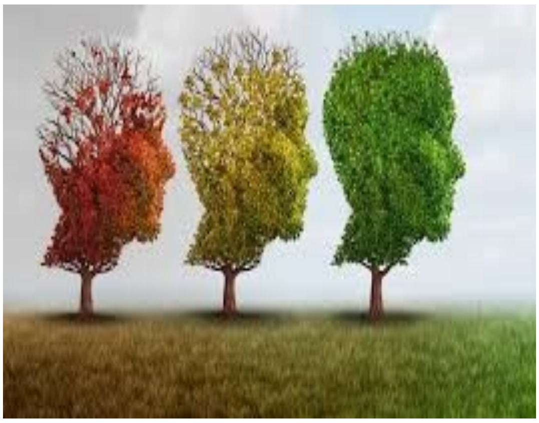

2. **Supportive hearing intervention for dementia patients:** which aims at recruiting participants with memory and hearing impairment. There is evidence that improving hearing function in elderly people may represent a potentially reversible cause of cognitive impairment, or may optimize remaining cognitive and functional ability and improve quality of life for patients and caregivers. In this trial intervention includes: clinical assessment and correction of hearing in an audiology and ENT clinic, fostering adherence with hearing aids: patient focus, knowledge, awareness and communication skills about dementia and hearing impairment: care partner focus and recap/ review.

3. "**Cognitive Stimulation therapy (CST) for individuals with Neuro Cognitive Disorders (Dementia): A feasibility Study from Pakistan- SAHARA.** The aim of the proposed project is to explore the feasibility of CST techniques for people with dementia in Pakistan. CST comprises of wide range of activities like Life history, current affairs, orientation, visual clip, physical games etc. Research evidence has shown that it not only improves cognition through engagement but also improves mood, quality of life, communication skills and social interactions of people with dementia.

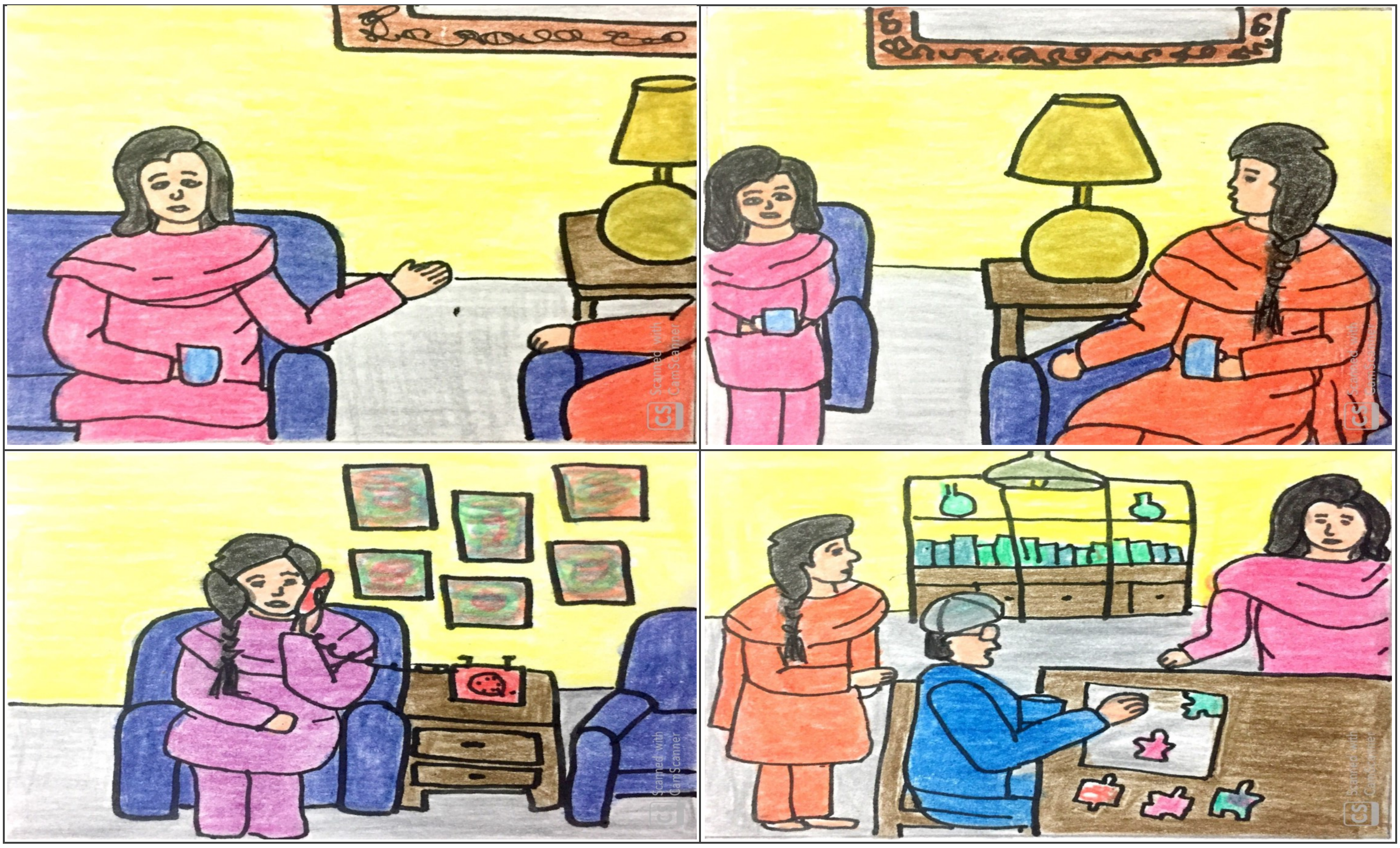

Pakistan Institute of Living and Learning

Capacity Building and Social Media Coverage

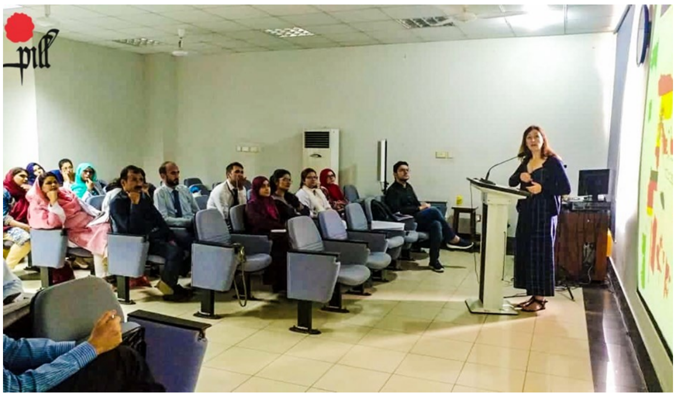

Karachi: Dr. Laoise Renwick lecture at Dow University of Health Sciences

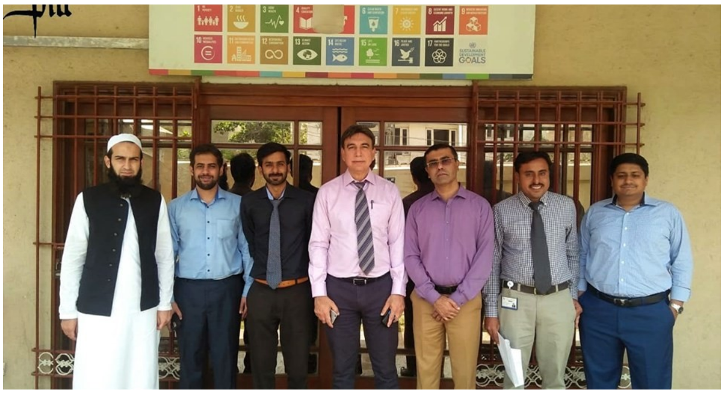

Karachi: Collaboration with Sustainable Development Goals (SDGs) Support Unit Sindh

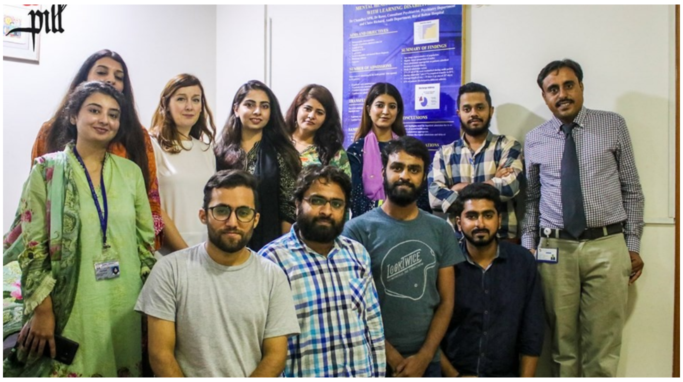

Karachi: Discussion Group on Psychological Problems among Youth

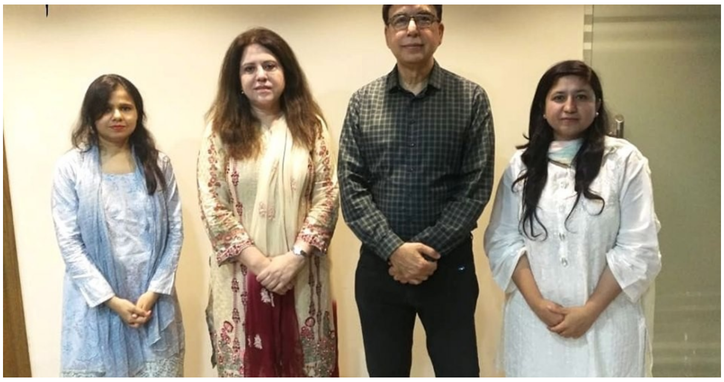

Islamabad: Collaboration meeting with Dr. Samra Deputy Programs Director for non communicable diseases & mental health

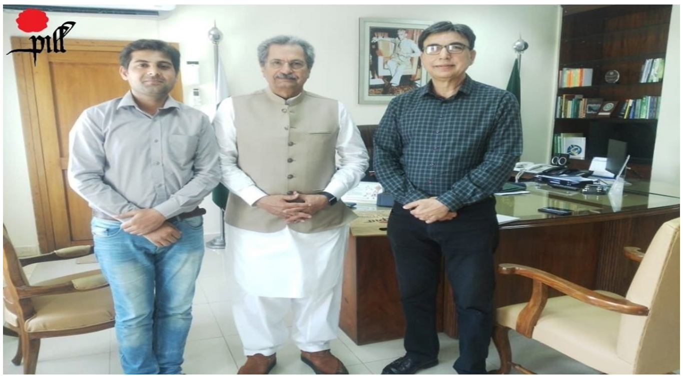

Islamabad: Collaboration meeting with Federal Education Minister Shafqat Mahmood

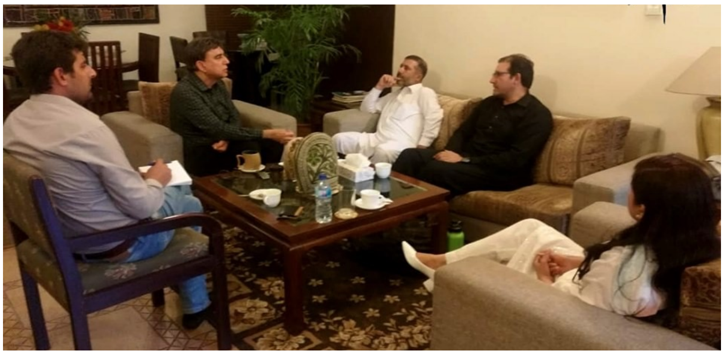

Islamabad: Collaboration meeting with Dr Karim Khan, Head of Department of Economics PIDE

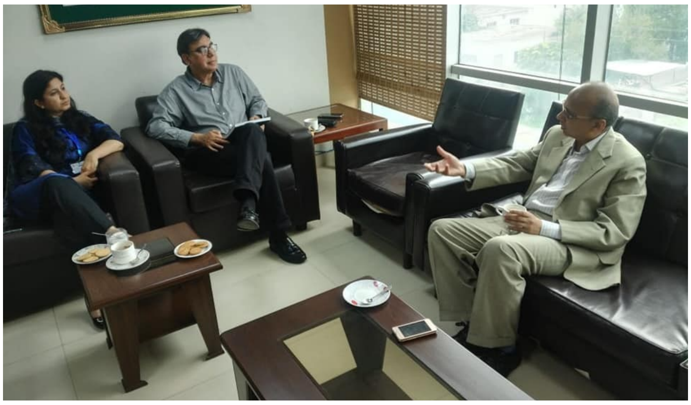

Islamabad: Collaborative meeting with Chief Health Officer Dr. Muhammad Asif

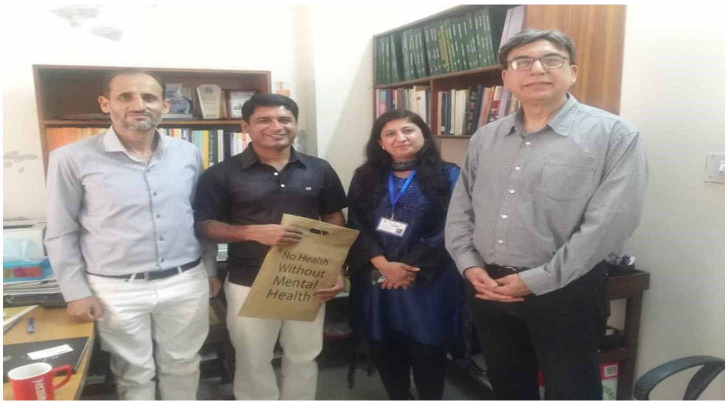

Islamabad: Visit to Institute of Psychology, Quaid-e-Azam University

Pakistan Institute of Living and Learning

Upcoming Capacity Building Events at Pakistan Institute of Living & Learning

(for further details visit www.pill.org.pk)

Pakistan Institute of Living and Learning

WORLD ALZHEIMER DAY 21st SEPT 2019

MANCHESTER GLOBAL FOUNDATION

CALL FOR POSTER COMPETITION

Dementia in South Asia

- **Pakistan Institute of Living and Learning (PILL) in collaboration with University of Manchester (GCRF) and Global Brain Health Institute calls upon young inspiring artists to submit drawings that dwell on the issue of dementia in South Asia**
- **To mark world Alzheimer day, we are looking for creative ideas to help develop awareness and understanding on the topic of dementia in South Asia.**
- **Art and humanities give us the ability to think critically and creatively, to reason and ask questions.**

Pakistan Institute of Living and Learning

GRANT WRITING RETREAT

Learn how to write a winning grant application

October 2019

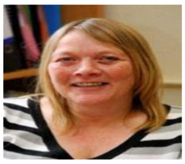

Professor Karina Lovell
Professor of Mental Health
University of Manchester

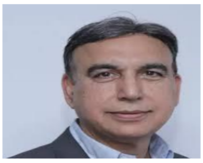

Professor Nusrat Husain
Professor of Psychiatry
Director Global Mental Health
University of Manchester

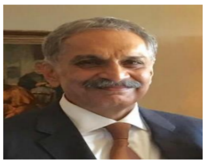

Professor Imran B Chaudhry
Professor & Chairman of Psychiatry, Ziauddin Hospital Karachi

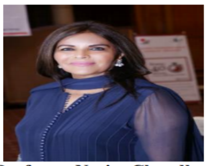

Professor Nasim Chaudhry
Professor of Psychiatry
Dow University of Health Sciences

Pakistan Institute of Living and Learning is keen to provide support to researchers and this is a fantastic opportunity for those who need some time and space to concentrate on a grant they have in mind. Join PILL for a grant application writing training and gain a greater understanding on what funders are looking for in an application and learn more about the range of grants that maybe available to you.

INTERNSHIP PROGRAMME

CAPACITY BUILDING IN MENTAL HEALTH

For those who want to enhance their skills in the field of mental health

Pakistan Institute of Living and Learning

No Health Without Mental Health

Substance Use Disorders : Assessment, Treatment and Continuum of care

Certificate in the Management of Substance Use Disorder

Total Credit Hours: 72

Schedule
Classes twice a week (Saturday & Sunday)

Monthly Fee
Rs. 22,500/- only

Level 1
Introduction to Substance Use Disorder

Level 2
Comorbidity & Assessment of Specific Population with SUD's

Level 3
Recovery and Continuum of Care for Substance Use Disorder

MANCHESTER GLOBAL FOUNDATION

Pakistan Institute of Living and Learning

No Health Without Mental Health

Psycho Social Intervention : A Mental Health Approach

Certificate Program for Health Professionals

Total Credit Hours: 62

Schedule
Classes twice a week (Saturday & Sunday)

Monthly Fee
Rs. 10,000/- only

Level 1
Fundamentals of Mental Health

Level 2
Understanding of Common mental Health Problems

Level 3
Approaches of Psycho-Social Intervention

MANCHESTER GLOBAL FOUNDATION

Specially For
Nurses, Midviveries & Social Workers

Pakistan Institute of Living and Learning

Our Collaborating Institutes

PILL Offices

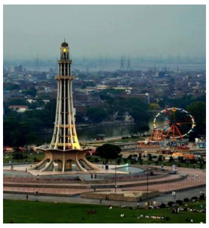
Lahore: New Muslim Town, Wahdat Road
042-35912278

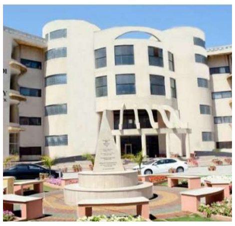
Rawalpindi: Institute of Psychiatry Rawalpindi, Benazir Bhutto Hospital
0322-4188265

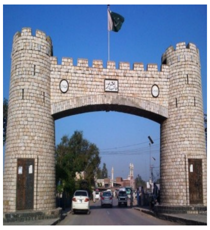
Peshawar: Sector F/3, Phase-6, Hayatabad
0348-0889909

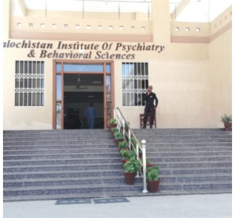
Quetta: Balochistan Institute of Psychiatry and Behavioural Sciences, Barwary Road
0308-3869545

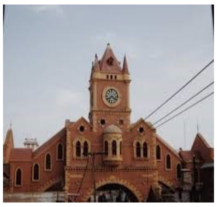
Hyderabad: Unit#7 Block-B, Latifabad
022-3408257

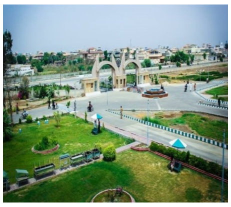
Nawabshah: Near Badshah Mazar, Ghareebabad
0334-2822133

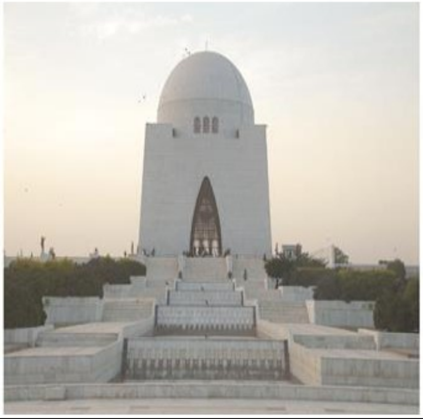

KARACHI (Head Office)

Address: D-9, BLOCK-I, North Nazimabad Town, Karachi
021 -36703712

11-C-6th Commercial Lane, Phase-5 Zamzama, Karachi (MCB street)
021-35871845, 0335-1024935

REMEDIAL CENTRE

HUSAIN REMEDIES

D-9, BLOCK-I, North Nazimabad Town, Karachi

Open 24 hours a day

Editorial Committee

Mrs. Afshan Qureshi, Uzma Omer, Sameen Ali, Usman Arshad, Amna Noureen, Farooq Ahmad, Shafaq Ijaz, Farhatulain, Marium Tahir, Mrs Rubina Tariq, Sanaullah, Mahnoor Saleem and PILL Team

Editorial Board

Ms. Sehrish Tofique, Mrs Rubina Tariq and Mr. Bilal Arif
