# Supplementary material for: Patient and Public Involvement for Dementia Research in Low- and Middle-Income Countries: Developing Capacity and Capability in South Asia
Source: Front Neurol. 2021 Mar 23;12:637000. doi: 10.3389/fneur.2021.637000 (PMC8021770; doi:10.3389/fneur.2021.637000)
Supplement: Supplementary file 1 [file Data_Sheet_1.zip › Supplementary File 6.docx]

**Supplementary file 6 – Dhaka survey additional responses**

1. If most people who need a hearing aid do not use one effectively, what are the reasons for ineffective use?

|  | **Reasons** | **Number of responses** |
| --- | --- | --- |
| 1 | Not fitting | 15 |
| 2 | Hard to use | 16 |
| 3 | Not tolerated | 29 |
| 4 | Too expensive | 5 |
| 5 | Lost/or broken | 0 |
| 6 | Not effective | 56 |
| 7 | Other (state) | 9 |
